# Supplementary material for: Parallel developmental changes in children’s production and recognition of line drawings of visual concepts
Source: Nat Commun. 2024 Feb 8;15:1191. doi: 10.1038/s41467-023-44529-9 (PMC10853520; doi:10.1038/s41467-023-44529-9)
Supplement: Supplementary file 1 — Supplementary Information [file 41467_2023_44529_MOESM1_ESM.pdf]

Supplementary Information for "Parallel developmental changes in children's production and recognition of line drawings of visual concepts"

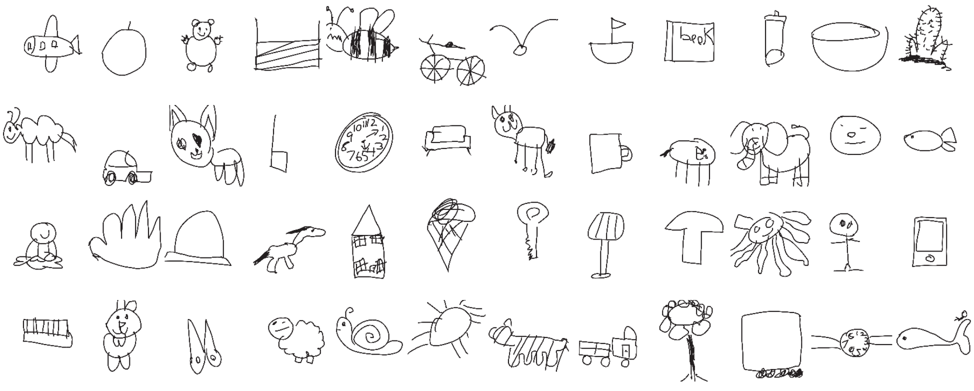

Supplementary Figure 1: **All drawing categories.** Examples of correctly classified drawings from each of the 48 categories presented at the experiment station in alphabetical order: airplane, apple, bear, bed, bee, bike, bird, boat, book, bottle, bowl, cactus, (2nd row): camel, car, cat, chair, clock, couch, cow, cup, dog, elephant, face, fish, (3rd row): frog, hand, hat, horse, house, ice cream, key, lamp, mushroom, octopus, person, phone, (4th row): piano, rabbit, scissors, sheep, snail, spider, tiger, train, tree, TV, watch, whale.

Age	Number of participants	Number of drawings
2-year-olds	1231	3651
3-year-olds	1402	5342
4-year-olds	1451	6559
5-year-olds	1189	6411
6-year-olds	878	4990
7-year-olds	660	3817
8-year-olds	478	2570
9-year-olds	309	1800
10+-year-olds	486	2630

Supplementary Table 1: **Participant demographics.** Number of participants and drawings included in the filtered drawing dataset by each age group.

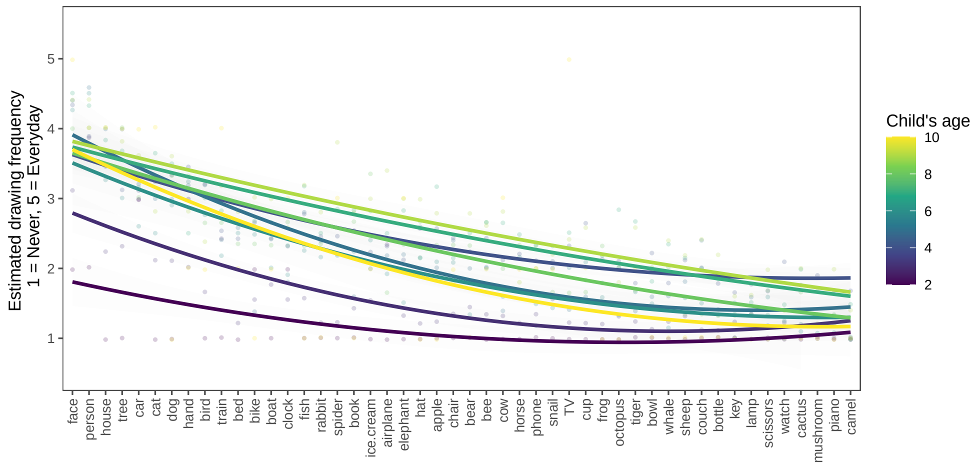

Supplementary Figure 2: **Category drawing frequency**. Frequency (y-axis) with which parents (recruited online) estimated their children drew each of the 48 categories in the dataset.

Age	Parental interference	Sibling interference	Num. participants
2-year-olds	53	44	3001
3-year-olds	118	35	2190
4-year-olds	105	33	1907
5-year-olds	45	21	1336
6-year-olds	17	20	1006
7-year-olds	22	18	784
8-year-olds	11	20	605
9-year-olds	8	8	401
10+-year-olds	15	26	567

Supplementary Table 2: **Reported interference.** Number of participants and types of interference reported in the initial dataset by each age group; all sessions where interference was reported were excluded from the filtered dataset.

	Estimate	Std. Error	z value	Pr(>|z|)	2.5 % CI	97.5 % CI
Intercept	-1.319	0.178	-7.410	<0.0001	-1.668	-0.970
Age (in years)	0.329	0.019	17.225	<0.0001	0.291	0.366
Est. drawing frequency	0.274	0.177	1.551	0.1209	-0.072	0.620
Avg tracing rating	0.279	0.020	14.320	<0.0001	0.241	0.318
Time spent drawing	0.195	0.019	10.065	<0.0001	0.157	0.233
Ink used	0.048	0.018	2.705	0.0068	0.013	0.083
Number of strokes	0.070	0.030	2.338	0.0194	0.011	0.129
Age*drawing frequency	0.029	0.014	2.030	0.0423	0.001	0.057

Supplementary Table 3: **Modeling drawing recognizability assessed by CLIP.** Model coefficients of a generalized linear mixed effect model (see *Methods*) predicting the recognizability of each drawing (i.e. binary classification scores) from CLIP classifications, including random intercepts for each category and participant.

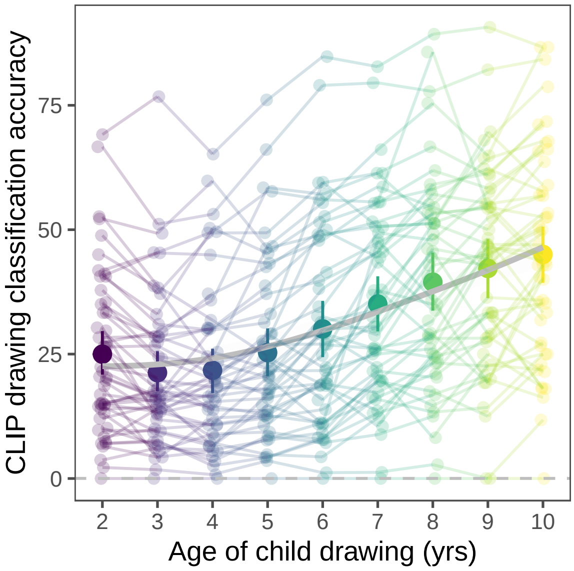

Supplementary Figure 3: **Drawing recognition with CLIP.** Drawing recognition accuracy, redone with CLIP classifications. Y-axis shows classification accuracy as a function of children's age (x-axis). Each dot represents data from an individual category, which are connected by individual trend lines. Error bars represent bootstrapped 95% confidence intervals across all 48 categories; all N=37770 drawings were included.

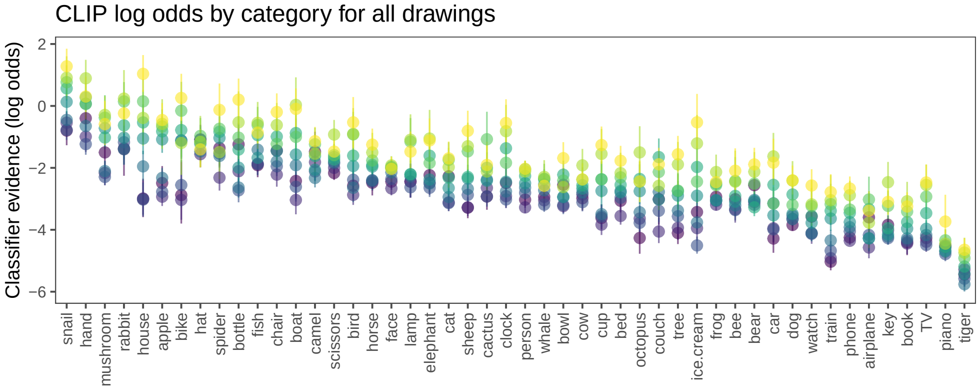

Supplementary Figure 4: **Classifier evidence using CLIP.** CLIP log-odds probabilities (y-axis) assigned to each category as a function of children's age; each dot represents data from an individual category and age. Error bars represent bootstrapped 95% confidence intervals across the number of drawings in each age/category bin; all N=37770 drawings were included.

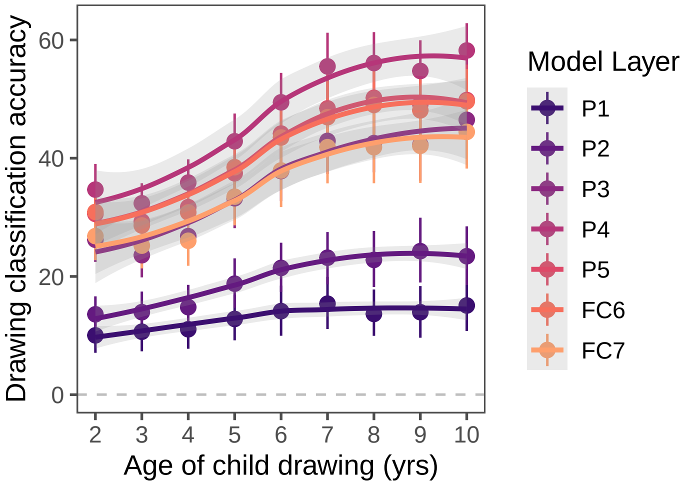

Supplementary Figure 5: **Drawing recognition across VGG-19 model layers**. Drawing accuracy as a function of children's age using embeddings from each layer in the VGG-19 network. Error bars represent 95 percent bootstrapped confidence intervals across all 48 categories in each age group.

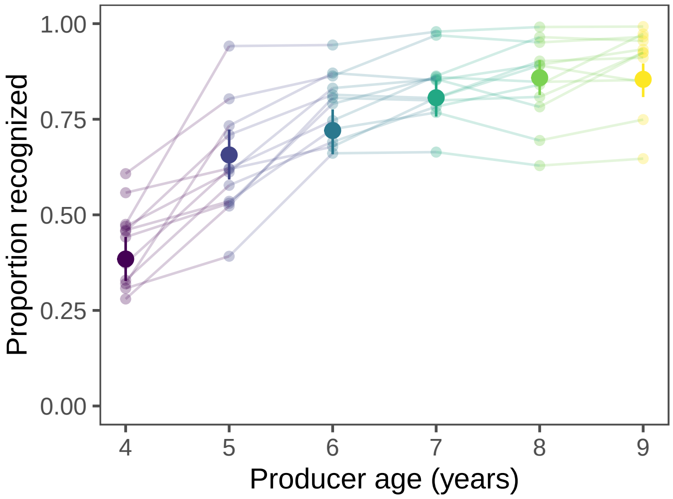

Supplementary Figure 6: **Drawing recognition in a controlled, experimental context.** Drawing recognition accuracy is plotted on the y-axis a function of children's age; children drew in response to verbal prompts in a controlled, experimental setting. Y-axis reflects the proportion of adult human observers who correctly identified the drawing in a 12AFC guessing task. Error bars reflect 95 percent bootstrapped confidence intervals across participants (N=121 total, approximately N=20 in each age group).

	Estimate	SE	df	t	Pr(>|t|)	2.5% CI	97.5% CI
Intercept	0.736	0.024	12.390	30.226	<0.001	0.688	0.784
Est. drawing frequency	0.043	0.024	9.853	1.833	0.097	-0.003	0.090
Age (in years)	0.151	0.011	247.928	14.348	<0.001	0.131	0.172
Age*drawing frequency	0.007	0.007	1282.435	1.073	0.283	-0.006	0.021

Supplementary Table 4: **Modeling drawing recognition in a controlled, experimental context.** Model coefficients of a linear mixed-effect model predicting the recognizability of each drawing (here, proportion of adults who recognized a drawing as assessed by crowd-sourced adult behavioral data, see *Methods*) as a function of children's age and estimated drawing frequency. All predictors are z-scored and random intercepts for each category and participant are included.

	Estimate	Std. Error	z value	Pr(>|z|)	2.5 % CI	97.5 % CI
Intercept	-0.965	0.237	-4.065	<0.0001	-1.430	-0.500
Age (in years)	0.360	0.021	17.006	<0.0001	0.318	0.401
Freq in adult books	0.515	0.312	1.653	0.098	-0.096	1.126
Age-of-Acquisition	0.702	0.418	1.678	0.093	-0.118	1.521
CHILDES frequency	0.129	0.398	0.323	0.747	-0.652	0.910
Drawing frequency	-0.290	0.326	-0.889	0.374	-0.930	0.349

Supplementary Table 5: **Analyzing drawing recognition by frequency metrics.** Model coefficients of a generalized linear mixed-effect model predicting the recognizability of each drawing (i.e. binary classification scores) from children's age, the frequency of each category in CHILDES (data for 91.8 percent of categories), the estimated Age-of-Acquisition using WordBank data (data for 73 percent of categories), and the frequency of each word in adult English books. All predictors are z-scored; random intercepts for each category and participant were included.

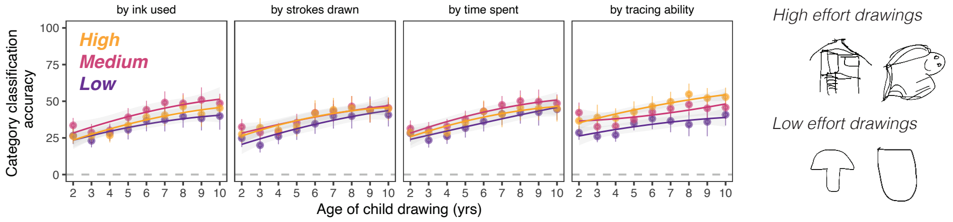

Supplementary Figure 7: **Effects of effort and visuomotor control on drawing recognition.**(Left): Classification accuracy by age, split into bins according to whether children expended a greater/lesser amount of strokes, ink, or time, and by their estimated tracing abilities (see Methods). (Right): Example drawings where children spent higher/lower amounts of *effort*—greater/lower than average number of strokes, time spent drawing, or 'ink' used.

	Term	VIF	SE Factor
1	Age (in years)	1.26	1.12
2	Estimate drawing Frequency	1.00	1.00
3	Average tracing score	1.24	1.11
4	Drawing duration (s)	1.30	1.14
5	'Ink' used	1.29	1.14
6	Number of strokes	1.07	1.03
7	Age*Estimated drawing frequency	1.01	1.00

Supplementary Table 6: **Multicolinearity analysis.**Results of the multicolinearity analysis for the predictors used in the main GLMM predicting the recognizability of children's drawings.

	Estimate	SE	df	*t*	Pr(>|t|)	2.5 % CI	97.5 % CI
Intercept	-0.687	0.107	43.356	-6.434	<0.001	-0.896	-0.478
Age (in years)	0.111	0.015	3544.182	7.354	<0.001	0.082	0.141
Est. drawing frequency	0.020	0.114	42.904	0.174	0.86	-0.204	0.243
Avg tracing rating	0.101	0.015	3964.435	6.753	<0.001	0.071	0.130
Time spent drawing	-0.019	0.018	7710.462	-1.060	0.29	-0.054	0.016
Ink used	0.018	0.017	7888.865	1.042	0.30	-0.016	0.052
Number of strokes	0.063	0.018	8110.693	3.535	<0.001	0.028	0.098
Age*drawing frequency	0.011	0.013	7672.028	0.811	0.42	-0.016	0.037

Supplementary Table 7: **Modeling changes in children's correctly recognized drawings.** Model coefficients of a linear mixed effect model predicting the log-odds probability assigned to correctly classified drawings using VGG FC6 embeddings (see *Methods*). All predictors were z-scored; the model includes random intercepts for each category and participant.

	Estimate	SE	df	*t*	Pr(>|t|)	2.5% CI	97.5% CI
Intercept	0.139	0.092	45.902	1.517	0.136	-0.041	0.318
Age (in years)	0.181	0.018	3577.121	10.167	<0.001	0.146	0.216
Est. drawing frequency	-0.109	0.096	45.592	-1.138	0.261	-0.298	0.079
Avg tracing rating	0.049	0.017	4262.357	2.806	0.005	0.015	0.083
Time spent drawing	0.069	0.020	9331.202	3.554	<0.001	0.031	0.108
Ink used	0.067	0.019	9520.800	3.635	<0.001	0.031	0.104
Number of strokes	0.026	0.020	9927.340	1.314	0.189	-0.013	0.066
Age*drawing frequency	-0.035	0.015	9693.984	-2.324	0.020	-0.064	-0.005

Supplementary Table 8: **Modeling changes in children's correctly recognized drawings using CLIP.** Model coefficients of a linear mixed effect model predicting the log-odds probability assigned to correctly classified drawings using CLIP embeddings, including random intercepts for each category and participant. All predictors were z-scored.

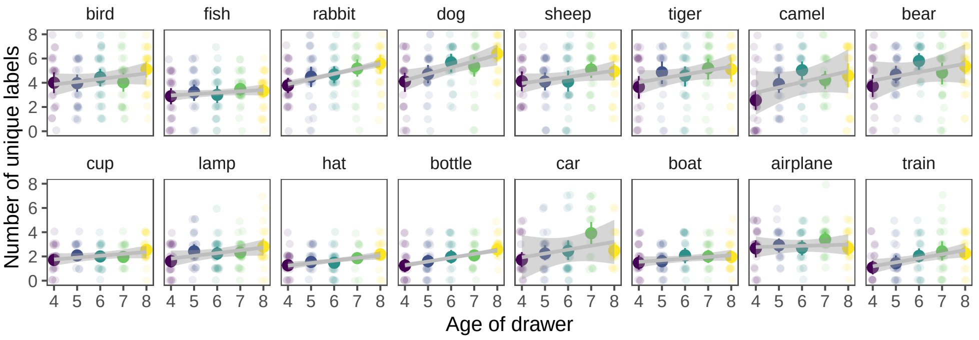

Supplementary Figure 8: **Unique parts included in children's drawings across development.** Number of unique parts included in each drawing for all object categories included in the semantic part annotation subset (N=2,088 drawings of 16 categories) across age; each dot represents an individual drawing, error bars represent 95% confidence intervals across drawings.

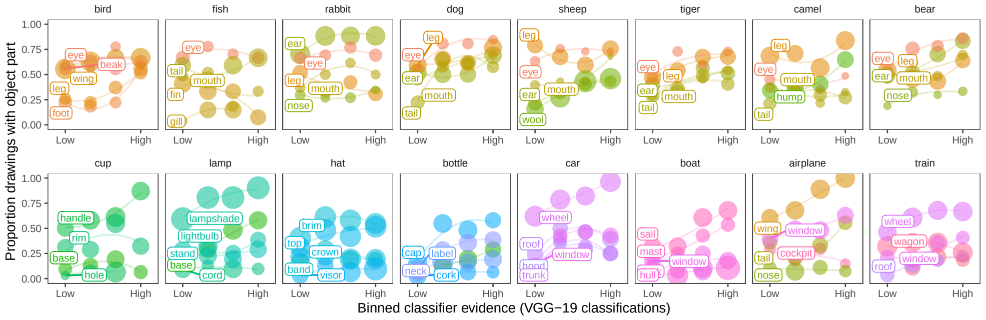

Supplementary Figure 9: **Object part information varies with model classifier evidence.** Object parts included for each category as a function of the VGG-19 classification evidence, binned into quartiles; only the top 4 object parts that were frequently included (excluding head/body) are shown here for visualization purposes. Dot size represents the visual emphasis on each part (proportion of ink allocated to a given object part).

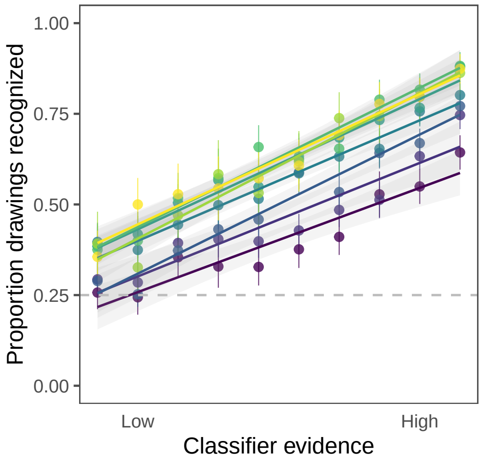

Supplementary Figure 10: **Drawing recognition modeled using CLIP classifier evidence.** Replication of the main interaction on visual recognition behaviors (proportion recognized, y-axis) by recognizer age (individual lines colored by age) and classifier evidence, here using CLIP classification probabilities (binned into deciles on the x-axis).

	Estimate	SE	z value	Pr(>|z|)	2.5 % CI	97.5 % CI
Intercept	0.397	0.183	2.167	0.03	0.038	0.757
Classifier evidence	0.987	0.215	4.590	<0.0001	0.566	1.409
Recognizer age	0.367	0.021	17.644	<0.0001	0.326	0.408
CLIP evidence*Recognizer Age	0.091	0.018	5.079	<0.0001	0.056	0.126

Supplementary Table 9: **Drawing recognition modeled using CLIP classifier evidence.** Model coefficients of a generalized linear mixed-effects model predicting binary visual recognition performance for each drawing as a function of recognizer age and CLIP classifier evidence in each drawing that was recognized by children. All predictors were z-scored prior to analysis such that coefficients are comparable. All significance tests are Wald significance tests based on the coefficient values; these tests are two-tailed.

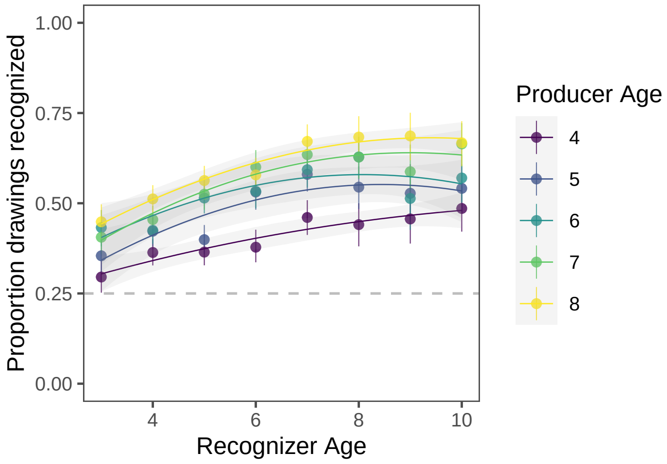

Supplementary Figure 11: **Drawing recognition modeled by the age of the child who produced the drawing.** Proportion of drawings recognized (y-axis) as a function of both the age of the child participating (x-axis) and the age of the child who originally produced the drawing (each line represents a different age). Error bars depict 95% bootstrapped confidence intervals across participant means (N=1789 children total).

	Estimate	SE	z value	Pr(>|z|)	2.5 % CI	97.5 % CI
Intercept	0.668	0.099	6.715	<0.0001	0.473	0.863
Classifier evidence	0.518	0.051	10.057	<0.0001	0.417	0.618
Recognizer age	0.141	0.023	6.190	<0.0001	0.096	0.185
Classifier evidence*Recognizer Age	0.056	0.023	2.464	0.014	0.011	0.101

Supplementary Table 10: **Drawing recognition modeled in high-performing children.** To ensure that these results were not driven by differences in motivation or general task performance, we also conducted our main analyses on a very restricted subset of our participants. We excluded any participant that did not achieve 100% on the photograph matching trials or that scored less than 50% on the drawing recognition trials. While this excluded nearly two-thirds of our participants, there were nonetheless N=649 participants in this subset. Above shown are model coefficients of a generalized linear mixed-effects model predicting binary visual recognition performance for each drawing as a function of recognizer age and classifier evidence in each drawing that was recognized by children. All predictors were z-scored prior to analysis such that coefficients are standardized and comparable. All significance tests are Wald significance tests based on the coefficient values; these tests are two-tailed. No adjustments were made for multiple comparisons.

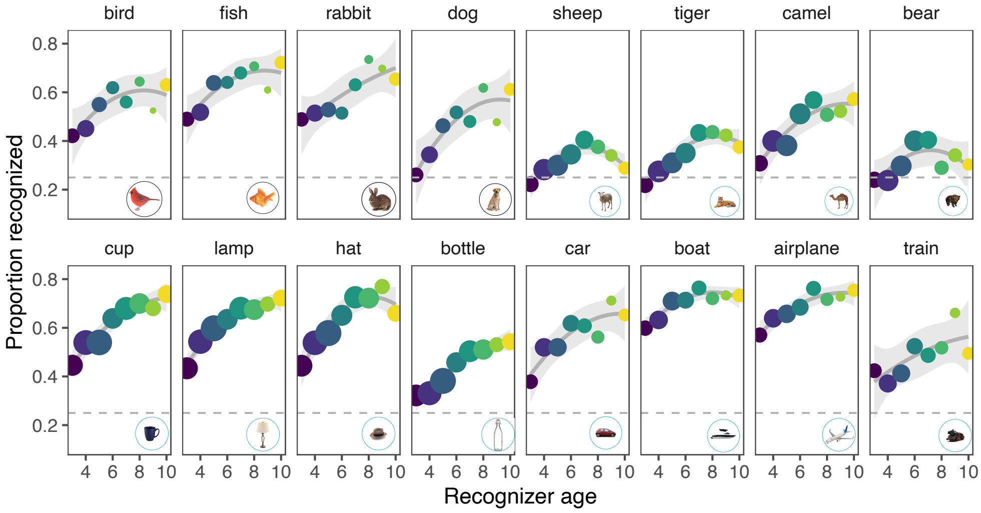

Supplementary Figure 12: **Drawing recognition by category.** Children's drawing recognition behavior for each of the 16 categories included in the recognition games; categories are grouped by the respective 4AFC game they were embedded in. Dots are scaled by the amount of data available from each age for each category (younger children were more frequent participants). Photo icons for each category are shown in the bottom right of each panel.

	Estimate	SE	z value	Pr(>|z|)	2.5 % CI	97.5 % CI
Intercept	0.047	0.202	0.233	0.82	-0.349	0.443
Number of unique parts	129.392	6.097	21.221	<0.0001	117.442	141.342
Number of unique parts^2	-34.984	2.944	-11.882	<0.0001	-40.755	-29.213
Recognizer Age	0.340	0.020	17.046	<0.0001	0.301	0.379
Number of unique parts x Recognizer Age	12.697	2.537	5.004	<0.0001	7.724	17.670
Number of unique parts^2 x Recognizer Age	-8.948	2.213	-4.043	<0.0001	-13.285	-4.610

Supplementary Table 11: **Modeling children's drawing recognition by part inclusion.** Above shown are model coefficients of a generalized linear mixed-effects model predicting binary visual recognition performance for each drawing as a function of recognizer age and number of unique parts in each drawing that was recognized by children. All predictors were z-scored prior to analysis such that coefficients are standardized and comparable. All significance tests are Wald significance tests based on the coefficient values; these tests are two-tailed. Random slopes were included for the number of parts included in each drawing; random intercepts were included for each recognizer.

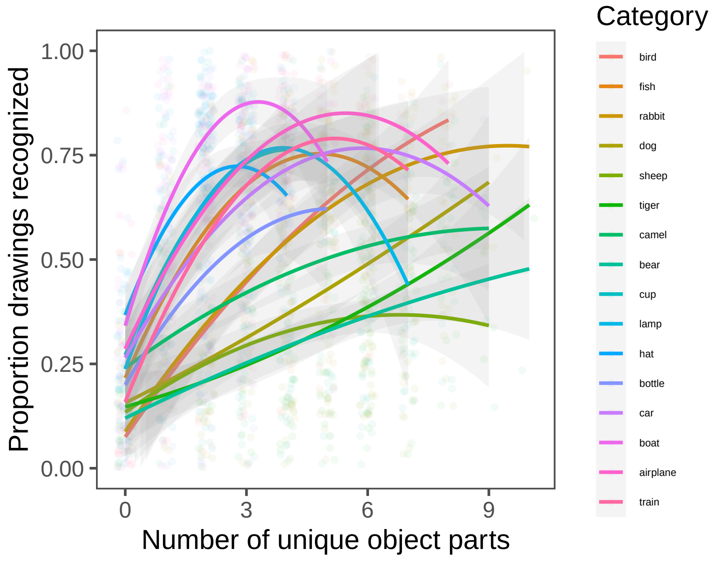

Supplementary Figure 13: **Drawing recognition for each category by number of unique parts.** Drawing recognition for each category as a function of the number of unique parts included in each drawing; each individual dot is a unique drawing.
