# Supplementary material for: Parallel developmental changes in children’s production and recognition of line drawings of visual concepts
Source: Nat Commun. 2024 Feb 8;15:1191. doi: 10.1038/s41467-023-44529-9 (PMC10853520; doi:10.1038/s41467-023-44529-9)
Supplement: Supplementary file 3 — Reporting Summary [file 41467_2023_44529_MOESM3_ESM.pdf]

nature portfolio

Corresponding author(s): Bria Long, NCOMMS-21-15973B

Last updated by author(s): 10/23/2023

Reporting Summary

Nature Portfolio wishes to improve the reproducibility of the work that we publish. This form provides structure for consistency and transparency in reporting. For further information on Nature Portfolio policies, see our Editorial Policies and the Editorial Policy Checklist.

Statistics

For all statistical analyses, confirm that the following items are present in the figure legend, table legend, main text, or Methods section.

n/a	Confirmed	
☐	☒	The exact sample size (*n*) for each experimental group/condition, given as a discrete number and unit of measurement
☐	☒	A statement on whether measurements were taken from distinct samples or whether the same sample was measured repeatedly
☐	☒	The statistical test(s) used AND whether they are one- or two-sided<br>*Only common tests should be described solely by name; describe more complex techniques in the Methods section.*
☐	☒	A description of all covariates tested
☒	☐	A description of any assumptions or corrections, such as tests of normality and adjustment for multiple comparisons
☐	☒	A full description of the statistical parameters including central tendency (e.g. means) or other basic estimates (e.g. regression coefficient) AND variation (e.g. standard deviation) or associated estimates of uncertainty (e.g. confidence intervals)
☐	☒	For null hypothesis testing, the test statistic (e.g. *F*, *t*, *r*) with confidence intervals, effect sizes, degrees of freedom and *P* value noted<br>*Give P values as exact values whenever suitable.*
☒	☐	For Bayesian analysis, information on the choice of priors and Markov chain Monte Carlo settings
☐	☒	For hierarchical and complex designs, identification of the appropriate level for tests and full reporting of outcomes
☒	☐	Estimates of effect sizes (e.g. Cohen's *d*, Pearson's *r*), indicating how they were calculated

Our web collection on statistics for biologists contains articles on many of the points above.

Software and code

Policy information about availability of computer code

Data collection	Code for displaying the experiments was written in Javascript, and all code is publicly available on the associated GitHub repository for this manuscript. https://github.com/brialorelle/drawing_production_and_recognition/
Data analysis	Code for analyzing the data were written in a combination of python and R; all code is publicly available on the associated GitHub repository for this manuscript. https://github.com/brialorelle/drawing_production_and_recognition/

For manuscripts utilizing custom algorithms or software that are central to the research but not yet described in published literature, software must be made available to editors and reviewers. We strongly encourage code deposition in a community repository (e.g. GitHub). See the Nature Portfolio guidelines for submitting code & software for further information.

Data

Policy information about availability of data

All manuscripts must include a data availability statement. This statement should provide the following information, where applicable:

- Accession codes, unique identifiers, or web links for publicly available datasets
- A description of any restrictions on data availability
- For clinical datasets or third party data, please ensure that the statement adheres to our policy

All filtered drawings, raw behavioral data from the recognition experiments, and all preprocessed data that support the findings are available at https://doi.org/10.17605/OSF.IO/QYMJ.

Research involving human participants, their data, or biological material

Policy information about studies with human participants or human data. See also policy information about sex, gender (identity/presentation), and sexual orientation and race, ethnicity and racism.

Reporting on sex and gender	Sex and gender information was not collected from the participants at the children's museum, and is not analyzed in this paper. Our goal was to facilitate participation at the kiosk at to require the least amount of information from participants as possible.
Reporting on race, ethnicity, or other socially relevant groupings	Race and ethnicity information was not collected from the participants at the children's museum, and is not analyzed in this paper. Our goal was to facilitate participation at the kiosk at to require the least amount of information from participants as possible.
Population characteristics	Our full age-breakdown is reported in the Appendix of the paper; younger children tended to participate more often at the kiosk that older children, likely due to the ages of children who tend to enjoy this particular Children's museum which has content skewed towards younger children (especially on the second floor of the museum, where this kiosk was located).
Recruitment	Participants were recruited via an engaging poster next to an iPad kiosk at the San Jose Children's Discovery Museum. Children and their parents who could not read English may have been less inclined to participate than others. No targeted recruitment was conducted other than placing the kiosk in the corner at the museum. Children whose parents/gaurdians did not have the means or time to bring them to this museum are not represented in this population.
Ethics oversight	The Institutional Review Board (IRB) at Stanford University approved this protocol (43992, Development of Drawing Abilities).

Note that full information on the approval of the study protocol must also be provided in the manuscript.

Field-specific reporting

Please select the one below that is the best fit for your research. If you are not sure, read the appropriate sections before making your selection.

☐ Life sciences ☒ Behavioural & social sciences ☐ Ecological, evolutionary & environmental sciences

For a reference copy of the document with all sections, see nature.com/documents/nr-reporting-summary-flat.pdf

Behavioural & social sciences study design

All studies must disclose on these points even when the disclosure is negative.

Study description	We collected drawing data and visual recognition data at a free-standing kiosk in a local children's science museum. Both studies were cross-sectional samples of children across a wide age range (2-10 years of age). Different versions of the drawing games and guessing games rotated at the station for several months at a time. These are quantitative data and analyses (even though drawings have been used in other qualitative studies).
Research sample	The research sample included children who came to the local children's science museum accompanied by their parents or guardians (who had to give consent for their child to participate and indicate their child's age in years); no other demographic information was collected. The kiosk was located in a quiet part of the museum and no additional recruiting was conducted. Instructions for parents could be displayed in four different languages (Chinese, Vietnamese, Spanish, or English) that the museum indicated are often spoken by the families who frequent the museum; however, children heard English during the experiment. This sample of children is likely somewhat representative of the children in the local surrounding area, but certainly not of the entire US or global population. This sample is more representative than other local children's museums (e.g., the Palo Alto Junior Museum & Zoo) with which the senior author has collaborations, but is ultimately a convenience sample. These limitations and directions for future work are noted in the General Discussion.
Sampling strategy	For drawing data, we aimed to collect as large of a dataset as possible to ensure generalizability across items and ages. As the museum is frequented by far fewer older children than younger children, the time at which we changed the items at the kiosk were determined by obtaining enough recognition/drawing data from older children. This was a convenience sample of children who voluntarily participated at the kiosk without our interventions. For the drawing data, we kept the set of categories running until we had at least 50 drawings from 9-10 year-olds. Given fluctuating attendance at the museum due to holidays and seasonal weather changes, we sometimes overshot these goals.<br><br>For the recognition experiments, we kept running the experiment until we had data from at least 30 9-10 year olds, checking every week; older children visited the station the least frequently and thus were the bottleneck on our data collection procedures. This was noted in our preregistration written after conducting the first two recognition games. No formal sample size calculation was performed, but we judged that this would be sufficient based on our initial piloting at the station and the observation that children completed an average of 20 trials per session.
Data collection	Drawing and guessing data were collected via an iPad Pro installed in a free-standing kiosk at a local children's museum (see photograph in Figure 1). The experimenter was not present during data collection. All data were recorded continuously and sent after each trial to our remote, secure server. The experimenters (i.e. the authors) were not blind to the hypothesis of the study.

Timing	We collected data continuously as children came to the museum, and we stopped collecting data due to closure of the museum due to the COVID-19 pandemic. We began drawing data collection in April of 2018 and ended data collection in March 2020. We began collection of recognition data in April 2019, and continued until March 2020.
Data exclusions	Given that we could not easily monitor all environmental variables at the kiosk that could impact task engagement (e.g., ambient noise, distraction from other museum visitors), we anticipated the need to develop robust and consistent procedures for data quality assurance. For drawing data, all drawings where interference from parents/siblings was reported were excluded from analyses. Raw drawing data were also screened for task compliance using a combination of manual and automated procedures (i.e., excluding blank drawings, pure scribbles, and drawings containing words), with an overall exclusion rate of 24.57% of the drawings and a final set of 37770 drawings from 8084 sessions. Please see methods for more details. For recognition data, we interspersed catch trials throughout the session, and we excluded participants who did not achieve at least 75% accuracy on these trials (N= 795) as well as 2-year-olds and adults, leaving us with a final sample of 1789 participants; these choices were registered after examining data from the first two recognition games.
Non-participation	N/A; we included data for any participant who advanced beyond the practice trials.
Randomization	For each drawing game session, the order of the category cues (i.e., "can you draw a [house]") was randomized; for each recognition game session, the order of the drawings presented was randomized, as was the order of the response buttons (see Methods for more details).

Reporting for specific materials, systems and methods

We require information from authors about some types of materials, experimental systems and methods used in many studies. Here, indicate whether each material, system or method listed is relevant to your study. If you are not sure if a list item applies to your research, read the appropriate section before selecting a response.

Materials & experimental systems

n/a	Involved in the study
☒	☐ Antibodies
☒	☐ Eukaryotic cell lines
☒	☐ Palaeontology and archaeology
☒	☐ Animals and other organisms
☒	☐ Clinical data
☒	☐ Dual use research of concern
☒	☐ Plants

Methods

n/a	Involved in the study
☒	☐ ChIP-seq
☒	☐ Flow cytometry
☒	☐ MRI-based neuroimaging

Plants

Seed stocks	n/a
Novel plant genotypes	n/a
Authentication	n/a
